# Assessment practices in undergraduate clinical medicine training: What do we do and how can we improve?

**DOI:** 10.4102/phcfm.v12i1.2341

**Published:** 2020-07-06

**Authors:** Hanneke Brits, Johan Bezuidenhout, Lynette J. van der Merwe, Gina Joubert

**Affiliations:** 1 Department of Family Medicine, School of Clinical Medicine, Faculty of Health Sciences, University of the Free State, Bloemfontein, South Africa; 2 Division of Health Sciences Education, Faculty of Health Sciences, University of the Free State, Bloemfontein, South Africa; 3 Undergraduate Programme Management, School of Clinical Medicine, Faculty of Health Sciences, University of the Free State, Bloemfontein, South Africa; 4 Department of Biostatistics, Faculty of Health Sciences, University of the Free State, Bloemfontein, South Africa

**Keywords:** assessment practices, clinical competence, improvement, undergraduate, South Africa

## Abstract

**Background:**

Assessment should form an integral part of curriculum design in higher education and should be robust enough to ensure clinical competence.

**Aim:**

This article reports on current assessment practices and makes recommendations to improve clinical assessment in the undergraduate medical programme at the University of the Free State.

**Methods:**

A descriptive cross-sectional study design was used. Qualitative and quantitative data were gathered by means of open- and closed-ended questions in a self-administered questionnaire, which was completed by teaching and learning coordinators in 13 disciplines.

**Results:**

All disciplines in the undergraduate medical programme are represented. They used different assessment methods to assess the competencies required of entry-level healthcare professionals. Workplace-based assessment was performed by 30.1% of disciplines, while multiple-choice questions (MCQs) (76.9%) and objective structured clinical examinations (OSCEs) (53.6%) were the main methods used during formative assessment. Not all assessors were well prepared for assessment, with 38.5% never having received any formal training on assessment. Few disciplines (15.4%) made use of post-assessment moderation as a standard practice, and few disciplines always gave feedback after assessments.

**Conclusion:**

The current assessment practices for clinical students in the undergraduate medical programme at the University of the Free State cover the spectrum that is necessary to assess all the different competencies required. Multiple-choice questions and OSCEs, which are valid and reliable assessment methods, are used frequently. Poor feedback and moderation practices should be addressed. More formative assessments, and less emphasis on summative assessment, should be considered. Workplace-based and continuous assessments may be good ways to assess clinical competence.

## Background

Assessment should form an integral part of curriculum design in higher education.^1^ Biggs explains that the outcomes of a programme, training and assessment should complement each other.^2^

**FIGURE 1:**
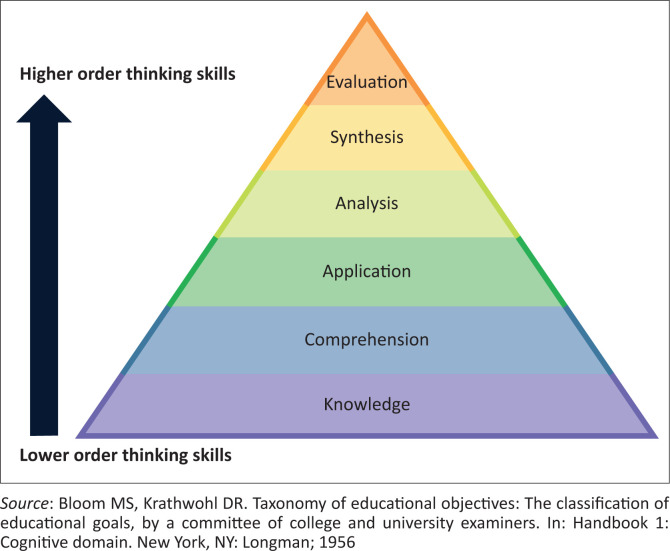
Bloom’s taxonomy.

The South African Qualifications Authority provides principles for credible assessment, among which are validity, reliability, fairness and practicability.^3^ Blueprinting is another important component of assessment, and ensures the reliability and validity of assessments.^4,5^ An assessment blueprint is a detailed plan (or table) of what is covered in the assessment.^4^ A blueprint should form part of the overall assessment planning and should include the content and cognitive levels that will be covered in the assessment process.^4^ The cognitive levels include knowledge, comprehension, application, analysis, synthesis and evaluation.^6^ These original levels as described by Blooms et al. are displayed in Figure 1.

The validity of assessments can be addressed using appropriate assessment methods and tools.^7^ Reliability is influenced by the quality and number of markers and questions, as well as the quality of assessment rubrics.^8,9^ To ensure that an assessment is practically feasible, resources, including assessors, patients, space, finances and equipment, should be considered when planning and performing the assessment.^4^

In outcomes-based curricula, such as medicine, the core competencies should be stated clearly in relation to the requirements of regulatory bodies.^10^ Moynihan et al.^11^ define core competencies as:

[*T*]he essential minimal set of a combination of attributes, such as applied knowledge, skills, and attitudes, that enable an individual to perform a set of tasks to an appropriate standard efficiently and effectively.

The Health Professions Council of South Africa (HPCSA) prescribes core competencies that should be incorporated in the training of undergraduate medical students in South Africa.^12^ These competencies were derived from the original Canadian Medical Society (CANMEDS) document^13^ and adapted for the South African context. The roles of a healthcare practitioner are central to these competencies: communicator, collaborator, health advocate, scholar, professional and leader, and manager.^12^

At the University of the Free State (UFS), a 5-year outcomes-based Bachelors of Medicine and Bachelors of Surgery (MBChB) programme is presented as training for medical doctors. The programme is divided into three phases over 10 semesters. Clinical training takes place in phase III (semesters 6–10). Clinical students rotate through six clinical blocks per year where they receive clinical and theoretical training. During rotations, continuous and end-of-block assessments take place. At the end of the academic year, students do a summative assessment in all disciplines. To progress to the next year or graduate (final year), students need to pass both the theoretical and practical component of each discipline separately. Knowledge, skills and attitudes are trained and assessed during this phase.^14^ This article is part of an overarching project to address the quality of undergraduate medical assessment. In other parts of the study, the students’ experiences and opinions were gathered, and the reliability of assessments was determined. Finally, lecturers discussed and made recommendations on how to improve current assessment practices to ensure defendable results.

The aim of this article was to report on current assessment practices in the clinical phase of the undergraduate medical programme at the UFS. The objectives were to describe different assessment methods that are used, the planning of the assessments, assessors and moderation practices, as well as how core competencies are assessed. Opinions on pass and fail decisions were also gathered, and recommendations for improving current assessments were obtained.

## Methods

A descriptive cross-sectional study design was used. Mainly quantitative data were gathered by means of a questionnaire and were supported by qualitative data that provide clarifying information and recommendations by participants.

### Study population and sampling

The study population consisted of teaching and learning coordinators (T&Ls) appointed in various clinical disciplines and module leaders of modules in disciplines that lacked T&Ls. The 13 clinical disciplines in phase III of the MBChB programme were eligible for inclusion.

A pilot study was conducted on two senior lecturers who were not part of the study population, to ensure that questions were clear and followed a logical sequence. Recommendations from a biostatistician were also incorporated before the questionnaire was finalised. One duplicate question was removed and the order of questions was changed to improve flow.

### Measurement

A questionnaire was developed, taking the principles of questionnaire development into account.^15^ Questions in the questionnaire were based on a framework to benchmark the quality of clinical assessment in a South African undergraduate medical programme.^16^

A self-administered, hard-copy questionnaire was distributed to T&Ls and/or module leaders in clinical disciplines at a phase III working group meeting. The staff members were invited to participate in the survey voluntarily. An information leaflet accompanied the questionnaire. Eligible staff members who were not present at the meeting received an electronic copy of the questionnaire, with an explanatory e-mail. An information leaflet and a hard copy of the questionnaire were also delivered to their offices. Participants returned questionnaires to the researcher in hard copy format or via e-mail. All participants signed informed consent. Data collection took place during September 2019.

The questionnaires obtained data about the different types or formats of assessment used, assessment planning and blueprinting, alignment of assessment with outcomes and training, the assessment of core competencies required by the HPCSA, moderation practices and recommendations for improving assessment. Clarification data on how the core competencies, as described by the Medical and Dental Board of South Africa (part of the HPCSA), are assessed were grouped per competency. In addition, suggestions and recommendations on how to improve assessment were obtained.

### Analysis of data

Data from the questionnaires were transferred to Excel datasheets by the researcher. The process of data transfer was done twice, to ensure integrity and accuracy. The Department of Biostatistics, Faculty of Health Sciences, did the data analysis of quantitative data with Analytics Software & Solutions (SAS) Version 9.4. Descriptive statistics, including frequencies and percentages, were calculated. Qualitative data were grouped by the first author according to themes.

### Ethical consideration

This study was approved by the Health Sciences Research Ethics Committee of the UFS (UFS-HSD 2019/0001/2304). Authorities at the UFS permitted the inclusion of UFS staff members in the study, and all participants signed informed consent. Although it was possible to identify individuals and disciplines from the questionnaires, no person or discipline was identified during the reporting of the data. All data were managed confidentially.

## Results

All 13 disciplines in the study population returned completed questionnaires: general surgery, internal medicine, paediatrics, obstetrics and gynaecology, psychiatry, family medicine, urology, orthopaedics, otorhinolaryngology, ophthalmology, oncology, nuclear medicine and anaesthesiology.

**FIGURE 2:**
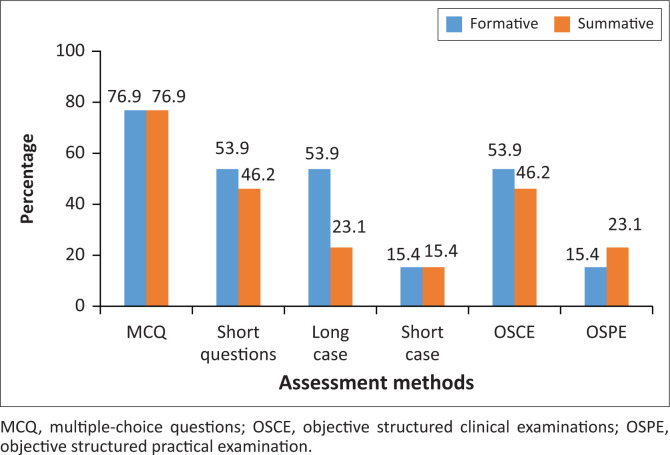
Percentages of different assessment methods used for formative and summative assessments.

Results show that different assessment methods were used for formative and summative assessment to assess theoretical knowledge and clinical skills, respectively. Workplace-based assessment (WBA), in the form of direct observation in the training area, was performed by 30.1% of disciplines. Multiple-choice questions (MCQs) were used for formative and summative assessments by 76.9% of disciplines. Objective structured clinical examination (OSCE) was used by 53.9% of disciplines for formative assessment and by 46.2% for summative assessment. More long cases were used for formative assessment than for summative assessment (53.9% vs. 23.1%), while more objective structured practical examination (OSPE) was used for summative assessments than for formative assessments (23.1% vs. 15.4%). Figure 2 displays the percentages of different assessment methods used for formative and summative assessments.

Current assessment practices were evaluated based on assessment factors, assessor factors and moderation and feedback. Most disciplines always (46.2%) or usually (38.5%) blueprinted their assessments. Resources were not taken into consideration in the planning of assessment by 15.4% of disciplines. Table 1 shows how often various assessment factors were taken into consideration during the planning of assessment.

**TABLE 1:** The use of various assessment factors for assessment planning (%).

Assessment factor	Always	Usually	Sometimes	Never
Blueprinting	46.2	38.5	0.0	15.4
Blooms taxonomy level	30.8	46.2	0.0	23.0
Alignment with teaching	53.9	46.2	0.0	0.0
Alignment with module outcomes	46.2	53.9	0.0	0.0
Resources (patients, assessors, finances, etc.)	61.5	23.1	0.0	15.4
Standardised assessment tools	38.5	30.8	7.7	23.0
Assessment dates	30.8	30.8	0.0	38.5

The results also show that assessors are not well prepared for the assessments in which they were involved: 38.5% had never received formal training before the assessment, while 30.8% had never been involved in assessment preparation. These results are shown in Table 2.

**TABLE 2:** Assessor factors that may influence the quality of assessment.

Assessor factor	Always	Usually	Sometimes	Never
Assessors received formal training	7.7	23.1	30.8	38.5
Assessors received informal training	38.5	30.8	7.7	23.1
Assessors may use subjective marking	23.1	23.1	7.7	46.2
Assessors are involved with the preparation of the assessment	7.7	30.8	30.8	30.8
Assessors agree on the tool before the assessment	15.4	53.9	7.7	23.1

Results also show that, in most disciplines, the practices of feedback and moderation were not well established. Only two disciplines (15.4%) always gave feedback after assessment, and only two disciplines made use of post-assessment moderation as a standard practice. Table 3 displays the results of feedback and moderation practices.

**TABLE 3:** Feedback and moderation practices.

Feedback and moderation	Always	Usually	Sometimes	Never
Students receive memorandums after assessment	15.4	23.1	7.1	53.9
Students receive feedback after assessment	15.4	38.5	23.1	23.1
Students receive results within 10 days of the assessment	53.9	7.7	15.4	23.1
Moderation takes place before the assessment	30.8	46.2	7.7	15.4
Moderation takes place after the assessment	15.4	38.5	23.1	23.1

Respondent comments regarding feedback and moderation included the following:

‘Moderation should not be a paper exercise. Moderators must help to improve the assessment.’

‘Our department needs to introduce post assessment moderation.’

**FIGURE 3:**
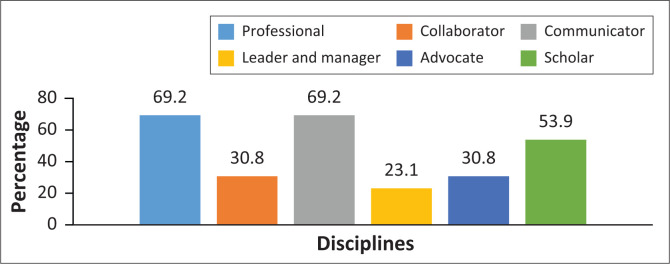
Percentages of disciplines that assessed the core competencies.

Most disciplines’ participants indicated that they assess some of the core competencies prescribed by the HPCSA. Figure 3 displays the percentages of disciplines that assessed the six core competencies.

Seven disciplines (53.8%) indicated that the clinical trainers assess professionalism during the rotation, while one department reported making use of patient feedback. One department presents a session on the core competencies. Collaboration was mainly assessed during interprofessional training sessions. Communication was not formally assessed in most disciplines. Two disciplines assessed communication formally during case presentations, and seven disciplines indicated that they assess it during case presentations as part of the overall assessment. One discipline reported assessing communication through referrals to other healthcare workers. Although 23.1% of disciplines indicated that they assess the competency ‘Leader and manager’, they did not indicate how this was done. Being a ‘Health advocate’ was assessed mainly through observation of patient–student contact. This included how the student manages resources, provides holistic care and develops alternative management plans. More than half the departments indicated that they assessed the core competency of ‘Scholar’ – they did this by considering preparation for assessments as sole element.

All disciplines (100%) regarded their assessments as fair, while 92.3% indicated that their assessments were reliable and of an appropriate standard. Two disciplines (15.4%) believed that their assessments were not appropriate for assessing knowledge and skills. Half the disciplines indicated that all students who passed were competent to register as entry-level healthcare practitioners, and 30.8% indicated that students who failed were not competent to register as entry-level doctors.

An open-ended question was posed to the respondents regarding suggestions on how to improve the current assessment. Recommendations centred on the types and process of assessment, integration and planning of assessment and resources. The following suggestions and recommendations were transcribed verbatim.

Types and process of assessment:

‘Maybe less emphasis on marks in the final cases and more formative assessment. Not all students perform best in high pressure clinical assessment.’

‘There is a need for alternative assessments. Simulated cases and formative assessment should be used.’

‘We need to change assessment procedures.’

‘Students that pass block assessments should not need to do the end of year assessment again. We should be able to declare them competent or not competent after a rotation.’

‘Patients change during assessment or change their story, it is not reliable.’

‘Standard setting and rubrics in clinical assessment can decrease subjectivity.’

Integration and planning of assessment:

‘What about one integrated OSCE?’

‘The whole department should be involved with training, setting of papers and assessment.’

‘We need to plan assessment from the beginning.’

Resources:

‘Lack of trained educators, staff and resources need to be addressed.’

‘Summative assessments are very labour intensive.’

‘The use of patients in summative assessment is problematic due to numbers (of assessments per day).’

‘We need expertise and support in IT [*Information Technology*]. We can’t spend so much time on this.’

To conclude the questionnaire, respondents could make final comments. All but one were regarding the Nelson Mandela Fidel Castro Medical Programme (NMFCMP) students who share the training platform with the UFS students. Their comments were:

‘The additional burden of the NMFCMP students have a negative impact on training and assessment. The extra number of students, as well as the extra effort to train them, decrease the student-lecturer ratios for current students.’

‘More students pose problems with patients and logistics during assessment.’

‘The current assessment is too labour intensive for the personnel numbers.’

‘The logistics with so many students is a nightmare. We need alternative assessment like workplace assessment.’

‘Resources should be addressed, like more lecturers and personnel to cope with these (NMFCMP) students.’

One final quote:

‘There is always scope for improvement, but we do good. If the clinical training is good, the assessment should confirm what you already know.’

## Discussion

Because the response rate was 100%, results are representative of assessment by clinical disciplines in the clinical phase of the undergraduate medical programme at the UFS. The results indicate the assessment practices in the different disciplines, while recommendations made reflect personal opinions of assessors responsible for assessments, and not necessarily that of the discipline involved.

As van der Vleuten^17^ states, ‘Any single assessment method can never be perfect on all criteria and in reality assessment always involves a compromise’. Therefore, different assessment methods should be used to assess students’ competence in clinical medical training. More than three-quarters of disciplines in this study used MCQs to assess theoretical knowledge. The advantage of using MCQs in assessment includes feasibility. In addition, when questions are well constructed, validity and reliability improve. Half the disciplines used short-written questions, which have the advantage that logic, reasoning and problem-solving can be assessed. The disadvantages of short-written questions include that the marking is labour intensive and leaves scope for subjective opinions and rater (marker) bias.^18^

Performance tests or assessments are used when learners need to demonstrate their competence and are appropriate for clinical medicine.^19^ These types of assessment reflect the level ‘does’ as described in Miller’s pyramid.^20^ Objective structured clinical examinations and WBA are examples of performance assessments. Workplace-based assessment is described as the observation of students while they are performing skills and competencies in the workplace (‘does’); they receive immediate feedback, to improve, reinforce or certify a skill.^21,22^ Less than one-third of disciplines in this study used direct observation or WBA for assessment. As WBA is authentic and tests performance,^21^ it is recommended that this method of assessment should play a bigger role in clinical assessment.^5^ Workplace-based assessment and training provide an opportunity to certify entrustable professional activities. An example of an entrustable professional activity is a student who demonstrates effective neonatal resuscitation every time it is done in the workplace; the skill can be certified, and there is no need for additional summative assessment.^23^ Half the disciplines used OSCEs as part of their assessment. Despite being labour intensive, the validity and reliability of OSCEs make it an excellent assessment method to measure clinical competence.^24^ Disciplines should be encouraged to use OSCEs to assess clinical competence. Half the disciplines used long cases during formative assessment. As a formative assessment method, observed long cases are appropriate for assessing holistic care, communication and problem-solving.^24,25^ A quarter of disciplines used unobserved long cases during summative assessment. This practice should be discouraged, as neither communication nor clinical skills can be assessed unless they are observed.^26,27^ During summative assessment, students do only two or three long cases, making this a less valid and reliable assessment method because of the small numbers.^28^

Constructive alignment is essential during curriculum planning.^1^ This involves the alignment of assessment with course outcomes and training.^2^ Part of this planning should also include blueprinting^4^ and determining the level of difficulty of the assessment using Bloom’s taxonomy.^6^ In this study, alignment between assessment, outcomes and training was always or usually taken into consideration during assessment planning. However, in less than half the assessments in the different clinical disciplines, blueprinting and the use of Bloom’s taxonomy were considered in assessment planning. In some disciplines, it was not done at all. This is an area where assessment can be improved.

The feasibility of an assessment is dependent on resources.^4^ However, resources were not consistently considered when assessment was planned. In general, the planning for assessment seems poor, with about a third of assessments performed without standardised assessments tools.

The reliability of an assessment is influenced by the assessment, the assessor and the student.^9^ In this study, assessors were not well prepared or trained for assessments. Less than a third of assessors always or usually received formal assessment training before assessments. Two-thirds of assessors had (always or usually) received informal training before the assessment, and less than half of assessors were usually involved with assessment planning. The above assessor factors may contribute to unreliable assessments. Resources and opportunities are available to address the lack of formal assessment training. Assessors should be encouraged and supported to attend these courses as part of professional development.

More than half the disciplines allowed subjective marking. For many years, objectivity had been regarded as a cornerstone of assessment – the introduction of MCQs made objectivity possible.^29^ In clinical practice, the management of patients is not unidimensional, and different approaches may all be correct. Ten Cate and Regehr^29^ argue that ‘subjective expert judgements by medical professionals are not only unavoidable but actually should be embraced as the core of assessment of medical trainees’.

Effective feedback in the workplace supports learning and competence development, which, in turn, improves patient care.^30^ For feedback to be effective, it should be focused, specific and on time.^31^ Feedback after assessment is a routine practice in only a few disciplines: only 15.4% of disciplines always gave feedback after assessments. If students do not receive feedback, they may continue to do things wrongly without knowing it. These poor feedback practices should be flagged and addressed, to improve clinical competence and ultimately patient care.^21,31^ Most disciplines indicated that they do not make memorandums available after assessments. This may be because many disciplines use MCQs and prefer to protect their question banks. Just more than half the disciplines always make marks available to students within the prescribed time frame of 2 weeks. When feedback and marks are late, students are already busy with a subsequent rotation and may not see the feedback as a learning opportunity.

From a quality assurance viewpoint, moderation of assessments is of utmost importance. Moderation is prescribed by the UFS assessment policy.^32^ Moderation usually or always takes place before assessment in at least 75% of disciplines, but only in half of disciplines after assessment. This is a missed opportunity to improve assessment and assessment practices.

Of the six core competencies prescribed by the HPCSA,^12^ only three – ‘Professional’, ‘Communicator’ and ‘Scholar’ – are assessed in more than half the disciplines. The competencies ‘Professional’ and ‘Communicator’ are assessed well during clinical training and patient presentations. ‘Collaborator’ is assessed mainly in disciplines where formal interprofessional training takes place. As students work daily with staff members from other disciplines, as well as in groups, the competency of ‘Collaborator’ can also be assessed through feedback from the team members. Although the competency ‘Scholar’ was assessed in most disciplines, it mainly took form of preparation for assessment (learning), and aspects such as the creation, dissemination and translation of knowledge were not assessed. Introducing a personal portfolio for each student, where all assessments of competencies throughout their training are recorded, may be effective in this regard.

Just less than half the T&Ls believed that some students who pass the summative assessment in clinical medicine are not competent to become entry-level healthcare practitioners fit for internship – they believe this despite assessors ‘certifying’ the students competent during assessment. This may be because of the phenomenon of failure-to-fail, where assessors pass incompetent students to prevent dealing with the consequences of failure.^33^ It may also happen because they are not confident in the quality of assessments.

Valuable recommendations were made on how to improve the quality of assessment. Most of the respondents suggested less emphasis on summative assessment with more formative assessments. A move towards WBA and formative assessment with feedback is recommended when clinical competence must be assessed.^30^ Better assessment planning, the use of standardised tools and better training of assessors were also proposed. Although these were individual recommendations, it will benefit to determine the need for training and provide support for all assessors.

The NMFCMP students are South African students trained in Cuba under the government to government agreement between South Africa and Cuba. These students train in Cuba and then return to South Africa where they are absorbed in the different undergraduate medical programmes to complete their last 18 months of training in South Africa.^34^ These students are included in the normal formative and end-of-block assessments of the universities. The increase in student numbers, with the added NMFCMP students, creates tremendous pressure on the current training platform with limited resources. This may be a risk for poor quality of assessment and need to be addressed.

The feedback from students, as well as a focus group interview with the T&Ls in the different modules, may assist to form a holistic picture of current assessment practices. This may also explore resistance or opportunities for assessor training. With this added information, a formal proposal for the improvement of undergraduate medical assessment can be made. For now, better implementation of moderation practices, specifically post-assessment moderation, will contribute to improved quality assurance.

## Conclusion

Current assessment practices for clinical students in the undergraduate medical programme at the UFS covers the spectrum that is necessary to assess all the different competencies. Multiple-choice questions and OSCEs, which are valid and reliable assessment methods, are used frequently. The lack of trained assessors, poor feedback and moderation practices should be addressed. More formative assessments, and less emphasis on summative assessment, should be investigated. Workplace-based and continuous assessment may be good ways to ensure the effective assessment of clinical competence.
